# Vaginal Laceration in an Open Pelvic Fracture Case Report: A Novel, Prophylactic Antibiotic Delivery Mechanism

**DOI:** 10.1155/2021/5594270

**Published:** 2021-05-03

**Authors:** Nishant Suneja, Eric H. Tischler, Skye Lockwood, Adam J. Wolfert, Daniel Martingano, Piyapa Praditpan, Thomas Lyon

**Affiliations:** ^1^Department of Orthopaedic Surgery, Harvard Medical School, Massachusetts General, 55 Fruit Street, Yawkey 3C, Boston, MA 02114, USA; ^2^Department of Orthopaedic Surgery and Rehabilitation Medicine, State University of New York, Downstate Medical Center, 450 Clarkson Avenue, MSC 30, Brooklyn, NY 11203, USA; ^3^University of Vermont, 184 S Prospect St., Burlington, VT 05401, USA; ^4^Department of Obstetrics & Gynecology, New York University Langone Hospital, 150 55th Brooklyn, NY 11220, USA; ^5^New York University School of Medicine, Department of Obstetrics & Gynecology, 150 55th, Brooklyn, NY 11220, USA; ^6^Department of Orthopedic Surgery, New York University Langone Hospital, 150 55th Street, Brooklyn, NY 11220, USA

## Abstract

**Introduction:**

Rotational displaced pelvic ring injuries are associated with internal injuries to both the gastrointestinal and genitourinary viscera and anatomic structures. Vaginal lacerations and open genitourinary-associated injuries are at increased risk of mortality due to sepsis. *Case Presentation*. This case presents a 65-year-old female status post-pedestrian-vehicle struck diagnosed with an open pelvic fracture with extension into the outer one-third of the vaginal wall. The patient was successfully treated with emergent surgical debridement, pelvic stabilization, and internal placement of a novel combination of metronidazole antibiotic gel and vancomycin/tobramycin Polymethyl methacrylate beads.

**Conclusion:**

No evidence of infection was observed with the use of topical metronidazole-coated vancomycin/tobramycin Polymethyl methacrylate beads for contaminated open pelvic fracture injury involving the vaginal wall. Further research on antibiotic gels for use in high-risk open fractures is required.

## 1. Introduction

Displaced pelvic ring fractures typically result from high energy impact associated with concomitant internal injuries to both pelvic and abdominal viscera [1–6]. Various classifications have been established to describe pelvic fracture patterns; however, the Young-Burgess classification is most commonly referenced [7]. This system uses radiographic findings of fracture and displacement patterns to aid in establishing the force vector in order to predict pelvic stability. The Young-Burgess contains four overall categories: lateral compression (LC), anteroposterior compression (APC), vertical shear (VS), and combined mechanisms [7].

Pelvic internal rotation deformities can cause significant medial displacement of the fractured hemipelvis, increasing the potential for laceration and/or puncture through both pelvic solid and hollow organs [8–10]. Injuries to the lower gastrointestinal (GI) tract, bladder, and genitourinary (GU) system have been well described [3, 4, 11]. Approximately, 4-5% of females with significantly displaced pelvic fractures incur a urethral injury [10]. Of these patients, 75-80% have concomitant vaginal laceration or tearing [10]. Vaginal lacerations caused by a pelvic fracture are associated with a significantly increased morbidity due to polymicrobial sepsis [12]. These injuries require emergent surgical debridement and broad-spectrum antibiotic coverage for both aerobic and anaerobic organisms.

## 2. Case Description

**Figure 1:**
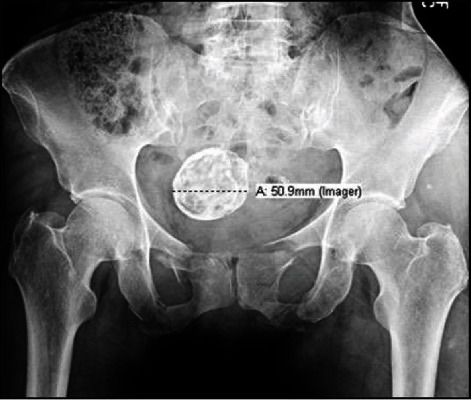
Initial anteroposterior pelvis X-ray showing bilateral pubic rami fractures and a large uterine fibroid, in a 65-year-old patient pedestrian struck by a motor vehicle.

A 65-year-old female presented to the ER within an hour of being pedestrian struck by a motor vehicle. The patient had a Glasgow Coma Scale (GCS) of 15 with an obvious right leg deformity. The patient reported both chest and abdominal pain. Upon further examination and radiographic studies, the patient was found to have an LC-I with an internal rotated pelvic ring injury, bilateral superior and inferior pubic rami fractures, and an impacted left sacral alar fracture. Additional polytraumatic injuries included the following: minimally displaced clavicle fracture, multiple rib fractures, pneumothorax, large 10 cm degloving injury of the right leg without bony involvement, and pelvic ring fractures. The patient was known to have a history of uterine fibroid which was visualized on the pelvic radiograph (Figure 1).

Additionally, the patient was also found to have vaginal blood present on digital exam. It was speculated that the pubic rami fracture fragment may have perforated through the vaginal wall.

**Figure 2:**
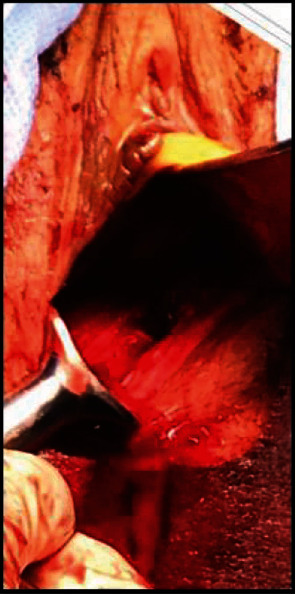
Picture showing the vaginal laceration through which the fractured bony fragment was assessed for irrigation and debridement.

**Figure 3:**
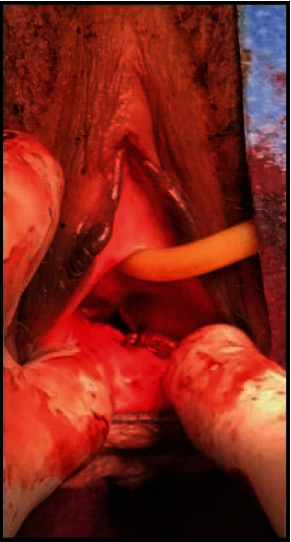
Primary closure of open vaginal wall injury.

Following initial trauma resuscitation and chest tube placement in the emergency room, the patient was transported to the operating room for (1) vaginal examination under anesthesia, (2) pelvic fracture stabilization, and (3) management of an extensive right lower extremity degloving injury. After intubation and general anesthesia, the patient received clindamycin and metronidazole for broad-spectrum perioperative antibiotic coverage and was placed in lithotomy position. OB/GYN performed vaginal examination and identified several lacerations: (a) 7 o'clock partial thickness tear; (b) 5 o'clock two-inch-deep, full-thickness laceration communicating with the left inferior pubic rami fracture (Figures 2 and 3); (c) 3 cm area of dead space surrounding the fractured edge.

The fractured rami was then excised through the vaginal wall tear by recreating the internal rotation force to the left lateral pelvic region. Formal irrigation and debridement (I&D) with pulse lavage were performed through the injured vaginal tissues.

**Figure 4:**
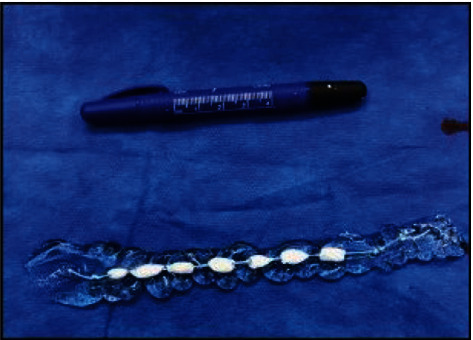
Surgeon made antibiotic-coated Polymethyl methacrylate (PMMA) beads surrounded by metronidazole gel.

Attention was addressed to the anterior pelvic fracture position. To obtain a more anatomic reduction to avoid further imposition of the fracture fragments into the central genitourinary region, an external rotation force was applied through direct pressure on bilateral ASIS and thighs. Prior to skin closure, 2 gm of vancomycin and tobramycin PMMA antibiotic PPMA beads covered with topical metronidazole gel was implanted into the pelvic dead space. The OB/GYN team performed closure of the vaginal wound and packed the vagina with a gauze roll coated with metronidazole gel, as per standard of care (Figure 4).

**Figure 5:**
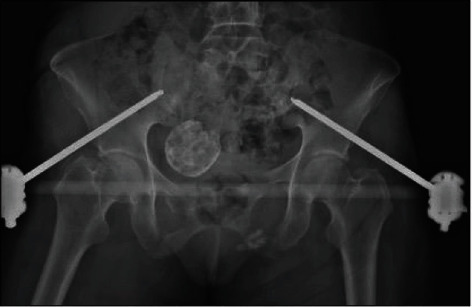
Anteroposterior pelvis radiograph showing the external fixator in place and the antibiotic beads surrounding the left inferior pubic rami fracture.

Under fluoroscopic guidance, a “Hannover style” pelvis external-fixator frame with two, 250 mm × 6 mm diameter pins in the Anterior Inferior Iliac Spine (AIIS) directed towards the Posterior Superior Iliac Spine (PSIS). A single anterior carbon fiber bar was applied with external fixation clamps. Final images demonstrated acceptable positioning of the pelvic ring and placement of antibiotic beads (Figure 5). Pin dressings were applied. The plastic surgery team performed an I&D and complex wound closure of the right lower extremity degloving injury. The patient was brought to the surgical intensive care unit for further trauma follow-up, requiring multiple flap repairs prior to hospital discharge. No signs of localized infection surrounding the pins or the vaginal laceration were observed. The patient remained hemodynamically stable, afebrile, with no leukocytosis during the hospital stay.

The patient maintained external fixation placement for 8 weeks (Figure 5), weight-bearing as tolerated (WBAT) on the right, and toe touch weight-bearing (TTWB) on the left. The decision for use of external fixation rather than performing internal fixation was a combination of mechanism of injury, the presence of multiple concurrent injuries, patient age, and risk of perioperative complications with additional surgery.

**Figure 6:**
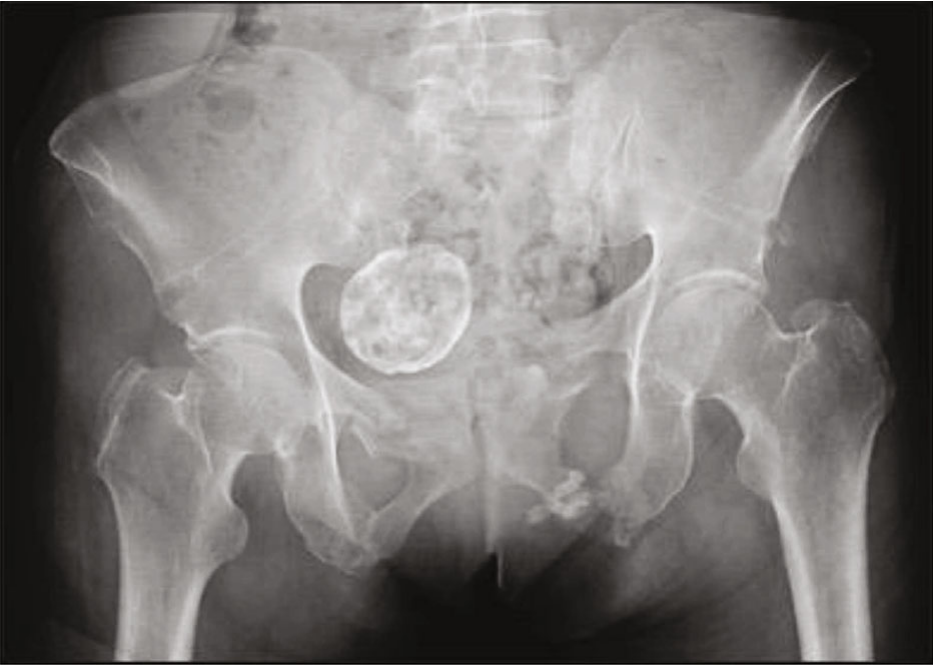
Anteroposterior pelvis radiograph after removal of the external fixator with healing of pubic rami fractures and presence of the antibiotic beads.

At two weeks, repeat radiographs demonstrated evidence of both bone healing and antibiotic beads (Figure 6).

At 2 months, the patient underwent external-fixator removal and bilateral hip manipulation under anesthesia. The patient was stable bilaterally and advanced to WBAT with an ambulatory assistive device. At one year, radiographs showed healed pubic rami fractures. The patient was ambulating well with minimal discomfort (Figure 6). No signs of superficial or deep infection from the open injury or discomfort from the in situ antibiotic beads.

## 3. Discussion and Review of the Literature

Open pelvic fracture management involves bleeding control, fracture stabilization, administration of appropriate antibiotics, and I&D of bone and soft tissue open injuries [11, 12]. Contaminated open pelvic lateral compression fractures will often require “recreation of injury” to excise debris/bone through the laceration used for debridement to protect the vaginal wall repair from reinjury and achieve adequate stabilization.

Associated concurrent GU injuries range between 6 and 15% of the cases [9, 11–13]. Although the bladder and urethra are internalized, sterile structures susceptible to injury from pelvic fractures. If either is ruptured or transected, it is considered “clean.” Contrastingly, “contaminated” vaginal wall injuries carry a very high risk of infection, especially if not initially recognized [10, 14, 15]. To better understand the management of these uncommon concurrent GU injuries, a literature search of pelvic fractures with associated GU injuries was conducted.

A total of 8 studies composing of 343 pelvic fractures and associated genitourinary injuries were identified (Table 1). The mortality rate among open pelvis fractures was fairly consistent at approximately 31% (range 20-40%) [1–9]. While the mechanism of injury varied among studies, motorcycle injuries were overwhelmingly responsible for the majority of high-energy open pelvic fractures. Black et al. reported among 52 open pelvic fracture cases over 2/3 were caused by either motorcycle (*n* = 15) or motor vehicle collisions (*n* = 20) [2].

Cannada et al. reported that among 21 women with open pelvic fractures, the vaginal laceration rate was 42.8% (9/21) [1]. This is consistent with previous literature reporting an average vaginal laceration injury rates of 34%. Furthermore, 14% of patients developed an infection (range: 6%-40%). It should be noted that a significant relationship between rectal tear, risk of infection, and mortality was observed [1]. Given the high risk of sepsis from these lacerations, practitioners must be diligent in their primary/secondary survey exam of any female with a high-energy pelvic fracture. Menstruating patients may require a formal OB/GYN evaluation if there is any doubt in this setting.

Despite the routine use of systemic antibiotics in these injuries, infections often complicate open pelvic fractures (Table 1) [1–9]. Topical application of antimicrobial gel to open fractures is well established in the literature given that the advantage of local antibiotic administration compared to parental administration is to maximize local concentration effect, while lowering undesirable systemic effects [12].

The most common clinically used delivery system for this local administration is Polymethyl methacrylate (PMMA) antibiotic beads [11–15]. These beads are often based on the same PMMA bone cements that are routinely used as prophylaxis in prosthetic joint infections in total joint replacement or revisions [11, 12]. Vancomycin/tobramycin antibiotic beads coated with metronidazole gel were utilized for our patient to optimize anaerobic coverage from the vaginal flora.

## 4. Conclusion

No evidence of infection was observed with the use of topical metronidazole-coated vancomycin/tobramycin PMMA beads for contaminated open pelvic fracture injury involving the vaginal wall. Furthermore, among 343 open pelvic fractures reported in the literature, there was a 31% mortality rate due to sepsis [1–9]. Among a subset of women over 65 with open pelvic fractures, 34% of sepsis cases were due to an internal laceration of the vaginal wall. This injury pattern is associated with a significant risk of deep pelvic sepsis, emphasizing the importance of an adequate vaginal exam for subtle findings of bleeding in any female presenting with a high-energy pelvic injury. The addition of an absorbable antibiotic gel such as “Metrogel” to antibiotic-impregnated PMMA beads may provide optimal coverage to prevent potentially lethal pelvic infections of aerobic and anaerobic organisms. Further research on antibiotic gels for use in high-risk open fractures is required.

## Figures and Tables

**Table 1:** A total of 8 studies composing of 343 pelvic fractures and associated genitourinary injuries.

Authors	Patients	Mortality rate (%)	Infection rate (%)	GU injury rate (%)	GI injury rate (%)	% female	Vaginal laceration rate (%)
Cannada et al.^∗^	64	23	6	17	31	32.8	43
Black et al.	52	19	N/A	8	27	17.3	56
Fu et al.	42	26	12	14	2	N/A	N/A
Giordano et al.	30	40	40	23	36	N/A	N/A
Dong et al.	41	24	17	29	37	N/A	N/A
Dente et al.	44	45	15	4	27	31.8	21
Perry et al.	31	42	16	39	N/A	29.0	11
Jones et al.	39	42	21	23	33	30.8	33
Total (weighted avg. %)	343	31	14	18	30	65	34

GI = gastrointestinal; GU = genitourinary; # = number; % = percent; ^∗^GI injuries listed as “abdominal injury”.

## Data Availability

Data supporting results comes from the patient medical record.
